# Postoperative bleeding after percutaneous transhepatic gallbladder drainage and aspiration in patients receiving antithrombotic therapy

**DOI:** 10.1371/journal.pone.0288463

**Published:** 2023-08-18

**Authors:** Takayuki Iwamoto, Takahiro Suda, Takanori Inoue, Yasutoshi Nozaki, Rui Mizumoto, Yuki Arimoto, Takashi Ohta, Shinjiro Yamaguchi, Yoshiki Ito, Hideki Hagiwara

**Affiliations:** Department of Gastroenterology and Hepatology, Kansai Rosai Hospital, Hyogo, Japan; Duta Wacana Christian University School of Medicine / Bethesda Hospital, INDONESIA

## Abstract

This study aimed to investigate the bleeding risk associated with percutaneous transhepatic gallbladder interventions in patients with acute cholecystitis receiving antithrombotic therapy. In this retrospective study, 194 consecutive patients who underwent percutaneous transhepatic gallbladder interventions for acute cholecystitis between April 2011 and April 2021 were enrolled. Patients were sorted into four groups: no prior antithrombotic therapy, discontinued antithrombotic drugs, single antithrombotic drug continued perioperatively, and multiple antithrombotic drugs continued perioperatively. The risk of postoperative bleeding after percutaneous transhepatic gallbladder interventions was evaluated via multivariate logistic regression analysis. Of the 116 (59.8%) patients receiving antithrombotic therapy, 32 (16.5%) discontinued antithrombotic drugs before their respective procedure, 50 (25.8%) continued a single antithrombotic drug, and 34 (17.5%) continued multiple antithrombotic drugs during the perioperative period. The rates of significant and severe bleeding were 10.3% (20/194) and 3.1% (6/194), respectively. The rate of significant bleeding was significantly higher in patients who continued multiple antithrombotic drugs than in patients who received no prior antithrombotic therapy (P = 0.006). In the multivariate logistic regression analysis, the continuation of multiple antithrombotic drugs during the perioperative period was a risk factor for significant bleeding after percutaneous transhepatic gallbladder interventions. In conclusion, the perioperative continuation of multiple antithrombotic drugs is a risk factor for postoperative bleeding after percutaneous transhepatic gallbladder interventions.

## Introduction

Increasing life expectancy has resulted in a growing population of older adults worldwide [1, 2]. Incidences of cardiovascular and cerebrovascular diseases, which are age-related and major causes of morbidity and mortality worldwide, are also on the rise. Antithrombotic drugs, including antiplatelets and anticoagulants, help prevent cardiovascular and cerebrovascular diseases [3, 4], with antithrombotic therapies growing in importance. However, antithrombotic therapy is a risk factor for bleeding complications. Multiple-drug antithrombotic regimens, such as dual antiplatelet therapy (DAPT), are prophylactic for cardiovascular and cerebrovascular diseases and are associated with increased bleeding events [5]. The increased risk of bleeding should be considered in patients receiving multiple-drug antithrombotic therapy when undergoing invasive procedures.

Acute cholecystitis (AC) is frequently encountered in general practice. In the Tokyo Guidelines 2018, which is a guideline for pathophysiology, diagnosis, and treatment of AC and acute cholangitis, urgent or early laparoscopic cholecystectomy was recommended in mild-to-moderate cases of AC that do not respond to initial conservative treatment. Percutaneous transhepatic gallbladder drainage (PTGBD) is recommended for patients with moderate-to-severe AC [6] who are at high risk for surgery. Percutaneous transhepatic gallbladder aspiration (PTGBA) is also performed as an alternative treatment for AC. Percutaneous transhepatic gallbladder interventions (PTGBIs), including PTGBD and PTGBA, are associated with bleeding complications. However, there is no consensus on the relationship between antithrombotic drugs and postoperative bleeding after PTGBI. A previous study showed that the continuation of antiplatelet drugs was a risk factor for severe bleeding after PTGBD [7]. In contrast, two studies reported that the administration of antithrombotic drugs did not increase the risk of bleeding complications [8, 9].

Few guidelines have focused on the perioperative administration of antithrombotic drugs before and after PTGBI [10, 11]. The Interventional Radiology Society (SIR) consensus guidelines recommend a discontinuation period for all antithrombotic drugs. However, this guideline is a consensus-based recommendation that lacks validation through data. Furthermore, it is often difficult to adhere to this recommendation in clinical practice since PTGBIs are often urgently required, or some patients with high thrombotic risk cannot discontinue all antithrombotic drugs. These patients, due to prior health conditions, cannot postpone procedures to sufficiently withdraw the antithrombotic therapy. Patients receiving multiple antithrombotic drugs often encounter these problems. Therefore, we studied the risk factors for postoperative bleeding after PTGBI associated with antithrombotic drug usage and discontinuation.

## Methods

### Patients

This study was approved by the Institutional Review Board of Kansai Rosai Hospital (Approval No. 22D014g) and conducted in compliance with the Declaration of Helsinki. The requirement for obtaining informed consent was waived owing to the retrospective nature of the study. We included 194 consecutive cases of PTGBI performed in 179 patients with AC between April 2011 and April 2021 at our institution. For patients who underwent multiple procedures during the same hospitalization, each procedure was analyzed independently. Using the electronic medical database, the following clinical data were collected: patient age; sex; history of diseases including advanced cancer, cardiovascular diseases, cerebrovascular diseases, hemodialysis, and liver cirrhosis; medication history; laboratory data (before and after the procedures) including hemoglobin, platelets, and the international normalized ratio of prothrombin time (PT-INR); bleeding events after the procedures; and perioperative thromboembolic events.

### PTGBI procedures

All procedures were performed under ultrasound (US) guidance. After securing the access route using US, color Doppler imaging was performed to confirm that no intervening blood vessels were present. For PTGBD, an 18-gauge needle was inserted into the gallbladder. The guidewire was coiled into the gallbladder, and a 7-Fr catheter was placed. For PTGBA, an 18 or 21-gauge needle was inserted into the gallbladder, as much infected bile as possible was aspirated, and the needle was removed immediately. At the end of all procedures, the absence of active bleeding or echo-free space was confirmed using color Doppler imaging.

### Management of antithrombotic therapy

Details of ongoing antithrombotic therapy were confirmed for all patients before the procedures. Antiplatelet drugs included aspirin, clopidogrel, prasugrel, and cilostazol, while anticoagulants included warfarin, rivaroxaban, dabigatran, apixaban, and edoxaban. The decision regarding the continuation or discontinuation of antithrombotic drugs was made based on the risk of thromboembolism associated with the withdrawal of the drugs. Some emergency procedures on patients refractory to initial treatment with antibiotics were unavoidably performed while continuing antithrombotic drugs. The drug withdrawal periods before and after the procedures were defined according to the SIR consensus guidelines: 3–5 days for aspirin, 5 days for clopidogrel, 7 days for prasugrel, 5 days until target PT-INR reached ≤1.8 for warfarin, two doses when creatinine clearance (CrCl) was ≥30 mL/min or three doses when CrCl was <15–30 mL/min for rivaroxaban, four doses (CrCl ≥50 mL/min) or six to eight doses (CrCl <30–50 mL/min) for dabigatran, four doses (CrCl ≥50 mL/min) or six doses (CrCl <30–50 mL/min) for apixaban, and two doses for edoxaban [10].

Patients were categorized into four groups according to their use of antithrombotic drugs: no prior antithrombotic therapy (no drug group), antithrombotic drugs discontinuation (discontinuing drug group), single antithrombotic drug continuation (single-drug group), and multiple antithrombotic drugs continuation (multiple-drugs group). Patients who did not use antithrombotic drugs regularly before the procedures were classified into the no-drug group, and those who discontinued antithrombotic drugs during the withdrawal period were classified into the discontinuing drug group. Patients who either received cilostazol as a single drug, replaced oral anticoagulant treatment with heparin, or received vitamin K to reverse warfarin effects were classified into the discontinuing drug group according to the SIR consensus guidelines [10]. Patients who continuously received one antithrombotic drug (except for cilostazol) were classified into the single-drug group. Those who continuously received multiple antithrombotic drugs, such as DAPT, triple antiplatelet therapy (TAPT), or a combination of antiplatelet drugs and anticoagulants, were classified into the multiple-drugs group. Patients who discontinued antithrombotic drugs for an insufficient amount of time were classified into the single-drug or multiple-drugs group according to the number of drugs taken.

### Definition of bleeding events

Significant bleeding events were defied as a decrease in hemoglobin levels >2 g/dL or apparent bleeding detected on imaging modalities after PTGBI procedures. Severe bleeding was defined as events requiring treatment, including red blood cell transfusions, transcatheter arterial embolization, or surgical procedures for hemostasis.

### Statistical analysis

Continuous variables are expressed as medians and ranges. Categorical variables are expressed as counts and percentages. The significant and severe bleeding rates were compared using Fisher’s exact test and the Mann–Whitney U test. Pairwise comparisons for groups with more than one participant were performed using Fisher’s exact test with Bonferroni correction for multiple comparisons.

The following factors were analyzed using univariate analysis: age, sex, procedures (PTGBD and PTGBA), advanced cancer, cardiovascular disease, cerebrovascular disease, hemodialysis, liver cirrhosis, platelet count, PT-INR, and regimens of antithrombotic therapy. Factors identified as statistically significant in the univariate analyses (P < 0.10) were analyzed using the multivariate logistic regression analysis model.

All statistical analyses were performed with EZR (Saitama Medical Center, Jichi Medical University, Saitama, Japan), which is a graphical user interface for R (The R Foundation for Statistical Computing, Vienna, Austria) designed to add frequently used statistical functions in biostatistics [12]. All P-values were derived from two-tailed tests, and P < 0.05 was considered statistically significant. Bonferroni correction was used for multiple comparisons.

## Results

Patient characteristics are presented in Table 1.

**Table 1:** Baseline characteristics of 194 patients that underwent percutaneous transhepatic gallbladder intervention.

Factors	All patients (n = 194)	PTGBD (n = 129)	PTGBA (n = 65)	P-value*
Age, years, median (range)	77 (43–98)	78 (46–98)	77 (43–97)	0.51
Sex				
male/female	108 (55.7)/86 (44.3)	69 (53.5)/60 (46.5)	39 (60.0)/26 (40.0)	0.45
Severity of cholecystitis				
mild/moderate/severe	47 (24.2)/108 (55.7)/39 (20.1)	33 (25.6)/69 (53.5)/27 (20.9)	14 (21.5)/39 (60.0)/12 (18.5)	0.70
Advanced cancer	44 (20.6)	34 (26.4)	10 (15.4)	0.10
Cardiovascular disease	97 (50.0)	58 (45.0)	39 (60.0)	0.07
Cerebrovascular disease	35 (18.0)	19 (14.7)	16 (24.6)	0.11
Hemodialysis	23 (11.9)	17 (13.2)	6 (9.2)	0.49
Liver cirrhosis	3 (1.5)	2 (1.6)	1 (1.5)	1.00
Antithrombotic therapy				
no prior antithrombotic therapy/single antithrombotic drug/multiple antithrombotic drugs	78 (40.2)/64 (33.0)/52 (26.8)	64 (49.6)/41 (31.8)/24 (18.6)	14 (21.5)/23 (35.4)/28 (43.1)	0.0001

Data are presented as n (%) unless otherwise stated.

*P-value between PTGBD and PTGBA.

Of the 194 patients who underwent PTGBIs, 108 were men, and 86 were women, with a median age of 77 years (range: 43–98). PTGBD was performed in 129 patients, while PTGBA was performed in 65 patients. Antithrombotic therapy at baseline was significantly different between the PTGBD and PTGBA groups (P = 0.0001). The percentage of patients receiving multiple antithrombotic drugs was higher in the PTGBA group than in the PTGBD group.

**Fig 1:**
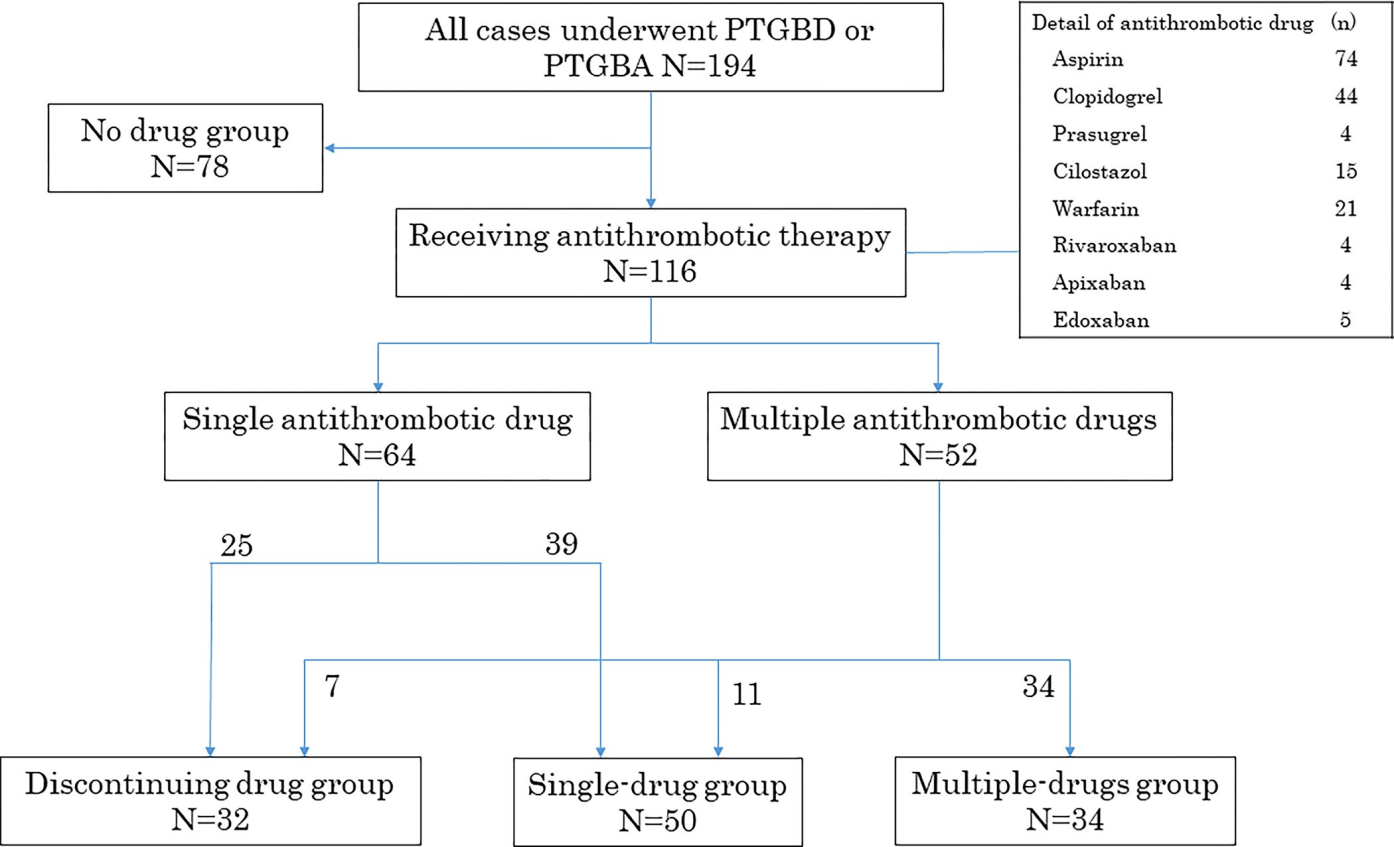
Flowchart of 194 patients who underwent PTGBD or PTGBA according to the administration of antithrombotic drugs. PTGBD: percutaneous transhepatic gallbladder drainage. PTGBA: percutaneous transhepatic gallbladder aspiration. No drug group: no prior antithrombotic therapy. Discontinuing drug group: antithrombotic drugs discontinuation. Single-drug group: single antithrombotic drug continuation. Multiple-drugs group: multiple antithrombotic drugs continuation.

Details of antithrombotic drugs at baseline and the management with antithrombotic drugs are shown in Fig 1.

Initially, 78 patients were in the no-drug group, 64 were treated with a single antithrombotic drug, and 52 were treated with multiple antithrombotic drugs. Antithrombotics were discontinued in 32 patients, single antithrombotic drug therapy was continued in 50 patients, and multiple antithrombotic drug therapy was continued in 34 patients. Four patients treated with cilostazol as the single drug, seven patients whose oral anticoagulant was replaced with heparin, and two patients who received vitamin K for reversal of warfarin were classified into the discontinuing drug group.

The overall rate of significant bleeding after PTGBI was 10.3% (20/194); the rates of significant bleeding for PTGBD and PTGBA were 10.1% (13/129) and 10.8% (7/65), respectively. No significant differences in significant bleeding were detected between these procedures (P = 1.00). No thromboembolism events occurred after discontinuation of antithrombotic therapy. The comparison of perioperative patient characteristics depending on the presence of significant bleeding after PTGBI is shown in Table 2.

**Table 2:** Comparison of perioperative patient characteristics associated with significant bleeding after percutaneous transhepatic gallbladder intervention.

Factors	Significant bleeding		P-value
	Present (n = 20)	Absent (n = 174)	
Age, years, median (range)	78 (45–89)	78 (43–98)	0.88
Sex (male/female)	11/9	97/77	1.00
Advanced cancer (present/absent)	6/14	38/136	0.41
Cardiovascular disease (present/absent)	15/5	82/92	0.03
Cerebrovascular disease (present/absent)	4/16	31/143	0.76
Hemodialysis (present/absent)	5/15	18/156	0.07
Liver cirrhosis (present/absent)	0/20	3/171	1.00
Platelet count, x10^4^/mL	15.6 (3.9–48.0)	18.3 (3.2–59.6)	0.04
PT-INR	1.19 (0.93–2.74)	1.21 (0.84–4.79)	0.65
Procedure (PTGBD/PTGBA)	13/7	116/58	1.00
Regimens of Antithrombotic therapy (no drug group/discontinuing drug group/Single-drug group/multiple-drugs group)	3/4/4/9	75/28/46/25	0.004

PT-INR: prothrombin time-international normalized ratio. PTGBD: percutaneous transhepatic gallbladder drainage. PTGBA: percutaneous transhepatic gallbladder aspiration. no drug group: no prior antithrombotic therapy. discontinuing drug group: antithrombotic drugs discontinuation. single-drug group: single antithrombotic drug continuation. multiple-drugs group: multiple antithrombotic drugs continuation.

**Fig 2:**
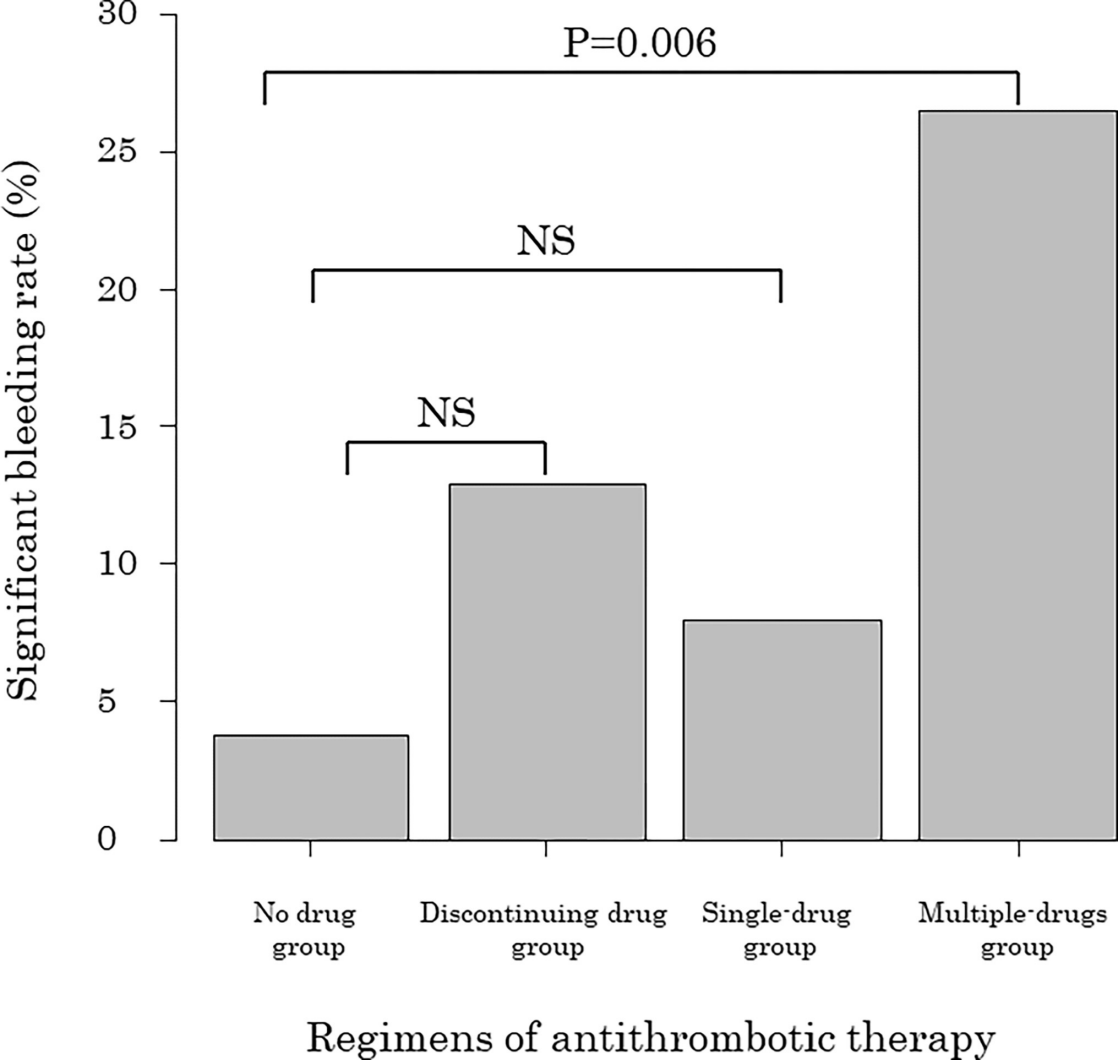
Comparison of significant postoperative bleeding after PTGBD or PTBGA according to the regimens of antithrombotic therapy. NS: not significant. no drug group: no prior antithrombotic therapy. discontinuing drug group: antithrombotic drugs discontinuation. single-drug group: single antithrombotic drug continuation. multiple-drugs group: multiple antithrombotic drugs continuation.

The presence of cardiovascular diseases, platelet counts, and regimen of antithrombotic therapy were significantly different between patients with and without significant bleeding after PTGBI. Antithrombotic therapy regimens were compared using Fisher’s exact test with Bonferroni correction (Fig 2).

Significant bleeding occurred in 3.8% (3/78) of patients in the no-drug group, 12.5% (4/32) of patients in the discontinuing drug group, 8.0% (4/50) of patients in the single-drug group, and 26.5% (9/34) of the patients in the multiple-drugs group. The rate of significant bleeding in the multiple-drugs group was significantly higher than that in the no-drug group (P = 0.006). The discontinuing drug group and the single-drug group were not significantly different from the no-drug group in this regard. In the univariate analysis, cardiovascular diseases, hemodialysis, and antithrombotic therapy regimens were significant factors influencing bleeding after PTGBI (P < 0.10). Multivariate analysis revealed that the continuation of multiple antithrombotic drugs was an independent risk factor for significant bleeding after PTGBI (P = 0.04) (Table 3).

**Table 3:** Risk factors associated with significant bleeding after percutaneous transhepatic gallbladder intervention.

Factors	Categories	Univariate analysis		Multivariate analysis	
		OR (95% CI)	P-value	OR (95% CI)	P-value
Age	≤80 years vs >80 years	0.86 (0.34–2.21)	0.75		
Sex	male vs female	0.97 (0.38–2.46)	0.95		
Advanced cancer	present vs absent	1.53 (0.55–4.26)	0.41		
Cardiovascular disease	present vs absent	3.37 (1.17–9.67)	0.02	2.04 (0.63–6.59)	0.23
Cerebrovascular disease	present vs absent	1.15 (0.36–3.69)	0.81		
Hemodialysis	present vs absent	2.89 (0.94–8.89)	0.06	1.70 (0.50–5.75)	0.39
Platelet count	≤15 x10^4^/mL vs >15 x10^4^/mL	0.73 (0.28–1.89)	0.52		
PT-INR	≤1.5 vs >1.5	3.54 (0.17–3.54)	0.73		
Procedure	PTGBD vs PTGBA	0.93 (0.35–2.45)	0.88		
Regimens of Antithrombotic therapy	Multiple-drugs group vs other groups	4.88 (1.83–13.00)	0.001	3.18 (1.05–9.58)	0.04

OR: odds ratio. CI: confidence interval. PT-INR: prothrombin time-international normalized ratio. PTGBD: percutaneous transhepatic gallbladder drainage. PTGBA: percutaneous transhepatic gallbladder aspiration. multiple-drugs group: multiple antithrombotic drug continuation. other groups: no prior antithrombotic therapy, antithrombotic drugs discontinuation, and single antithrombotic drug continuation.

The overall rate of severe bleeding after PTGBI was 3.1% (6/194). The rates of severe bleeding following PTGBD and PTGBA were 1.6% (2/129) and 6.2% (4/65), respectively. No significant difference in severe bleeding was detected between PTGBD and PTGBA (P = 0.10). The comparison of perioperative patient characteristics with respect to the presence of severe bleeding after PTGBI is shown in Table 4. Platelet counts and antithrombotic therapy regimens were significantly different between patients with and without severe bleeding after PTGBI (Table 4).

**Table 4:** Comparison of perioperative patient characteristics associated with severe bleeding after percutaneous transhepatic gallbladder intervention.

Factors	Severe bleeding		P-value
	Present (n = 6)	Absent (n = 188)	
Age, years, median (range)	78 (56–88)	77 (43–98)	0.99
Sex (male/female)	1/5	107/81	0.09
Advanced cancer (present/absent)	1/5	43/145	1.00
Cardiovascular disease (present/absent)	5/1	92/96	0.21
Cerebrovascular disease (present/absent)	2/4	33/155	0.30
Hemodialysis (present/absent)	2/4	21/167	0.15
Liver cirrhosis (present/absent)	0/6	3/185	1.00
Platelet count, x10^4^/mL	13.9 (3.9–17.3)	17.9 (3.2–59.6)	0.04
PT-INR	1.27 (1.02–2.74)	1.21 (0.84–4.79)	0.39
Procedure (PTGBD/PTGBA)	2/4	127/61	0.10
Regimens of Antithrombotic therapy (no drug group/discontinuing drug group/single-drug group/multiple-drugs group)	1/0/1/4	77/32/49/30	0.03

PT-INR: prothrombin time-international normalized ratio. PTGBD: percutaneous transhepatic gallbladder drainage. PTGBA: percutaneous transhepatic gallbladder aspiration. no drug group: no prior antithrombotic therapy. discontinuing drug group: antithrombotic drugs discontinuation. single-drug group: single antithrombotic drug continuation. multiple-drugs group: multiple antithrombotic drugs continuation.

In multiple comparisons of antithrombotic therapy regimens, the differences were not significant; however, the rate of severe bleeding in the multiple-drugs group was higher than that of the other groups (1.3% [1/78] in the no-drug group, 0% [0/32] in the discontinuing drug group, 2.0% [1/50] in the single-drug group, and 11.8% [4/34] in the multiple-drugs group). Univariate analysis revealed that the continuation of multiple antithrombotic drugs was a risk factor for severe bleeding after PTGBI (Table 5).

**Table 5:** Risk factors associated with severe bleeding after percutaneous transhepatic gallbladder intervention.

Factors	Categories	Univariate analysis	
		OR (95% CI)	P-value
Age	≤80 years vs >80 years	0.65 (0.12–3.62)	0.62
Sex	male vs female	0.15 (0.02–1.32)	0.09
Advanced cancer	present vs absent	0.67 (0.08–5.93)	0.72
Cardiovascular disease	present vs absent	5.22 (0.60–45.50)	0.14
Cerebrovascular disease	present vs absent	2.35 (0.41–13.40)	0.34
Hemodialysis	present vs absent	3.98 (0.69–23.00)	0.12
Platelet count	≤15 x10^4^/mL vs >15 x10^4^/mL	0.24 (0.04–1.35)	0.11
PT-INR	≤1.5 vs >1.5	3.77 (0.65–21.80)	0.14
Procedure	PTGBD vs PTGBA	0.24 (0.04–1.35)	0.11
Regimens of Antithrombotic therapy	Multiple-drugs group vs other groups	10.50 (1.85–60.10)	0.001

OR: odds ratio. CI: confidence interval. PT-INR: prothrombin time-international normalized ratio. PTGBD: percutaneous transhepatic gallbladder drainage. PTGBA: percutaneous transhepatic gallbladder aspiration. multiple-drugs group: multiple antithrombotic drugs continuation. other groups: no prior antithrombotic therapy, antithrombotic drugs discontinuation, and single antithrombotic drug continuation.

## Discussion

The management of antithrombotic drugs is important for reducing the risk of bleeding after invasive procedures. For surgery and endoscopic procedures, guidelines on the use of antithrombotic drugs has been widely discussed regarding patients who receive single or multiple antithrombotic drugs [13–17]. According to these guidelines, antiplatelet drugs, at least aspirin monotherapy should be continued during the perioperative period in patients with a high risk of thromboembolism.

PTGBIs are invasive procedures. Unlike in surgical and endoscopic procedures, bleeding following PTGBI is difficult to control due to the lack of direct visualization; thus, additional invasive procedures, such as embolization, may be required. Therefore, in patients receiving antithrombotic therapy, bleeding after PTGBIs should be given attention. Concerning PTGBI, several guidelines suggest perioperative management for patients receiving antithrombotic drugs [10, 11]. The SIR consensus guidelines propose an approach for periprocedural management of antithrombotic drugs in patients undergoing percutaneous imaging-guided interventions. However, the guideline is consensus-based to overcome the lack of evidence and is intended to serve as a reference when drafting guidelines for individual image-guided interventions [10].

Whether continuing antithrombotic therapy increases the risk of postoperative bleeding after PTGBI in patients with AC has not been directly assessed in randomized clinical trials. There is still a lack of data regarding the periprocedural management of patients receiving antithrombotic drugs, leaving several aspects of the relationship between PTGBI and postoperative bleeding unknown. First, few reports have described the risk of postoperative bleeding after PTGBI in patients receiving antithrombotic therapy [7–9]. In particular, only one study has evaluated the relationship between the use of multiple antithrombotic drugs and postoperative bleeding [7]. Sagami et al., in their systematic review citing results from these studies, noted that the bleeding risk following PTGBD in patients receiving antithrombotic drugs was controversial [18]. This is because conflicting results have been reported regarding whether antithrombotic drugs are risk factors for postoperative bleeding after PTGBD [7–9]. Second, the definition of patients receiving antithrombotic therapy differs among these reports. Hamada et al. defined the continued intake of antithrombotic drugs as patients receiving these drugs on the day of the procedure [7]. Dewhurst et al. defined continued intake of antithrombotic therapy as patients receiving antithrombotic drugs up to 5 days prior to a procedure [8]. Meanwhile, Shibasaki et al. discontinued antithrombotic drugs in all cases on the day they were diagnosed with AC [9]. Third, no reports include oral direct factor Xa inhibitors, which have been developed and administered to an increasing number of patients in recent years. Based on previous reports, we investigated whether the continuation of antithrombotic drugs increases postoperative bleeding after PTGBI, focusing on the continuation of multiple antithrombotic drugs, including DAPT or TAPT. In this study, the withdrawal period of antithrombotic drugs complied with the SIR consensus guidelines.

The significant bleeding rate of 10.3% (20/194) in this study was higher than the bleeding rate of 0–4.8% reported in previous studies [7, 19–23]. This difference may be due to the inclusion of mild-to-moderate bleeding events requiring no treatment or the higher percentage of patients receiving antithrombotic drugs in our study (59.8%) compared with that in previous studies (11.8–40.9%) [7–9]. The continuation of multiple antithrombotic drugs was a risk factor for significant and severe postoperative bleeding after PTGBI. There have been only three studies on the risk of postoperative bleeding after PTGBI in patients receiving antithrombotic therapy. Two studies concluded that PTGBD does not increase postoperative bleeding in patients receiving antithrombotic therapy [8, 9]. However, Hamada et al. [7] reported that continuation of antiplatelet drugs was associated with a significantly higher rate of severe bleeding compared with non-use, whereas discontinuation of these drugs carried a similar risk. In their study, anticoagulants did not significantly increase the risk of severe bleeding. Regarding the safety of continuing multiple antithrombotic drugs, only the study by Hamada et al. evaluated the relationship between multiple antithrombotic drugs and postoperative bleeding. In their report, 337 patients received dual therapy of antiplatelet drugs and anticoagulants. The rates of severe bleeding were 8% (2/25) in patients who continued both treatments, 15.8% (6/38) in patients who continued antiplatelet treatment but discontinued anticoagulants, 0% (0/9) in patients who discontinued antiplatelet treatment but continued anticoagulant treatment, and 2.3% (6/265) in patients who discontinued both drugs [7]. These results suggest that dual therapy with both antiplatelet drugs and anticoagulants may not be a risk factor for severe bleeding. However, in their study, the relationship between multiple antiplatelet therapies, including DAPT or TAPT, and severe bleeding was not described.

Based on the results of this study, we believe that whenever possible, PTGBI should be avoided in patients continuing the use of multiple antithrombotic drugs due to the significantly increased risk of postoperative bleeding. Meanwhile, patients with no prior antithrombotic drug usage of and those who discontinued the use of antithrombotic drugs based on the recommendation of the SIR guidelines had a similar risk for significant postoperative bleeding. Based on this, discontinuation of antithrombotic drugs for the period recommended by the SIR guidelines may be useful in reducing the risk of significant postoperative bleeding after PTGBI. However, in practice, patients with severe AC associated with delayed treatment or deteriorating general condition, or those at high risk for thromboembolism, often fail to comply with antithrombotic drug withdrawal according to the SIR consensus guidelines. The SIR consensus guidelines also state that the recommendations may not apply to emergency or urgent procedures where the risk associated with delayed treatment may outweigh the potential bleeding risk [10]. In fact, in this study, the complete discontinuation rate was 13.5% (7/52) for patients receiving multiple antithrombotic drugs, which was very low. Conversely, 21.2% (11/52) of patients receiving multiple antithrombotic drugs at baseline underwent PTGBI while continuation of a single antithrombotic drug, which was higher than that in those who completely discontinued drug intake (Fig 1). Herein, the risk of bleeding in the single-drug group was similar to that in the no-drug group. Therefore, we suggest that in patients receiving antithrombotic therapy with multiple drugs for whom complete discontinuation is difficult, reduction to a single drug may be an effective alternative to reduce the risk of postoperative bleeding.

Of the 34 patients in the multiple-drugs group in this study, 7 (20.6%) discontinued antithrombotic drug therapy 24 hours prior to the procedure; however, the withdrawal period did not meet the SIR consensus guidelines. The rates of significant and severe bleeding in patients with an incomplete withdrawal period were 28.6% (2/7) and 14.3% (1/7), respectively, which were similar to those of patients who continued multiple antithrombotic drug therapy (25.9% [7/27] and 11.1% [3/27], respectively) (data not shown). Owing to the small number of cases in this study, it is difficult to draw conclusions on whether discontinuing antithrombotic drugs 24 hours before the procedure reduces the risk of postoperative bleeding. It would be worthwhile to investigate the effects of the withdrawal of antithrombotic agents on postoperative bleeding rates in larger case studies using shorter withdrawal periods than those currently recommended in the SIR consensus guidelines. This may lead to the discovery of more feasible withdrawal periods for emergency or urgent procedures.

In recent years, the benefits and safety of endoscopic transpapillary gallbladder drainage (ETGBD) have been reported [24, 25]. ETGBD is useful for patients with coagulation abnormalities or thrombocytopenia, patients on antithrombotic medications, patients with ascites effusion, and patients whose anatomy makes percutaneous procedures difficult. It is also important to consider the indications for ETGBD in patients who are receiving multiple antithrombotic drugs and have difficulty discontinuing them.

Another noteworthy result of this study is the severe bleeding rate after PTGBA. PTGBA has two advantages over PTGBD. First, it can be performed in a simpler and quicker manner at the bedside using a US diagnostic device and a needle. Second, it has a similar or lower complication rate than PTGBD [19, 20]. Itoi et al. showed that the clinical success rate within 3 days of PTGBA was significantly higher than that of PTGBD. Additionally, the complication rate for PTGBA was lower than that for PTGBD in their study [26]. Although PTGBA is not recommended in the guidelines as a standard drainage technique for patients with AC, it is widely used as an alternative. Regarding PTGBA, severe bleeding is rare [19, 20, 27], and the rate of postoperative bleeding in patients on antithrombotic therapy has also not been investigated. The overall rates of severe bleeding were 6.2% and 1.6% in patients who underwent PTGBA and PTGBD, respectively; the rate of severe bleeding after PTGBD in our study was similar to that in previous studies [7, 19–23]. Although our sample size was small, the rate of severe bleeding after PTGBA in our study was higher than that in previous studies [19, 20, 27]. In our study, the percentage of patients who continued multiple antithrombotic drugs and underwent PTGBA (30.8%, 20/65) was significantly higher than that of those who underwent PTGBD (10.9%, 14/129). To avoid bleeding complications, PTGBA, which has reportedly lower complication rates than PTGBD, may have been chosen for high-risk patients. However, three of the four cases with severe bleeding after PTGBA had continued multiple antithrombotic drug therapy. Thus, the continuation of multiple antithrombotic drugs may have influenced the bleeding rates. In other words, PTGBA may not be advantageous regarding less postoperative bleeding in patients who continue receiving multiple antithrombotic drugs.

This study has few limitations. First, it was retrospective in nature, and the number of patients was small. A larger prospective study is needed to confirm our findings. Second, we did not evaluate technique-related data such as needle size, number of needle passes, and the experience of operators. Third, the small number of patients in this study receiving each type of antithrombotic drug and the large proportion of patients receiving multiple antithrombotic drugs made it difficult to assess in detail the association between each type of antithrombotic drug and the risk of postoperative bleeding, including dose-dependent effects of antithrombotic drugs. Finally, patients who were considered to be at a higher risk for procedure-related bleeding, based on laboratory data or the status of antithrombotic drugs, were more likely to undergo PTGBA. Hence, the rate of bleeding after PTGBD was potentially underestimated in our study.

In conclusion, the continuation of multiple antithrombotic drug therapy increases the risk of significant and severe bleeding after PTGBI. Discontinuation of antithrombotic drug therapy based on the withdrawal period recommended by the SIR consensus guidelines may reduce the postoperative bleeding rate.

## Supporting information

S1 Data Patient characteristics and data. (XLSX)
